# Successful treatment of cardiogenic shock due to Takotsubo syndrome with implantation of a temporary microaxial left ventricular assist device in transaxillary approach

**DOI:** 10.1186/s13019-023-02459-z

**Published:** 2023-11-27

**Authors:** Johanna K. R. von Mackensen, Ahmed El Shazly, Felix Schoenrath, Joerg Kempfert, Christoph T. Starck, Evgenij V. Potapov, Stephan Jacobs, Volkmar Falk, Leonhard Wert

**Affiliations:** 1 Department of Cardiothoracic and Vascular Surgery, Deutsches Herzzentrum der Charité – Medical Heart Center of Charité and German Heart Institute Berlin, Augustenburger Platz 1, 13353 Berlin, Germany; 2 DZHK (German Center for Cardiovascular Research) partner site Berlin, Berlin, Germany; 3 Department of Cardiothoracic Surgery, Charité − Universitätsmedizin Berlin, corporate member of Freie Universität Berlin, Humboldt-Universität zu Berlin, Berlin Institute of Health, Berlin, Germany; 4 Department of Health Sciences and Technology, ETH Zürich, Zurich, Switzerland

**Keywords:** Takotsubo, Cardiogenic shock, Impella, Temporary microaxial left ventricular assist device

## Abstract

**Objectives:**

Cardiogenic shock (CS) can occur in patients with Takotsubo syndrome (TTS). As TTS has received increasing attention and has been more closely researched, several aspects of the pathogenesis have been identified, particularly that an excessive release of catecholamines plays an important role. Nevertheless, evidence on specific therapy concepts is still lacking. As a result, TTS with severe hemodynamic instability and low cardiac output creates unique challenges, and mechanical circulatory support is needed with as few inotropic drugs as possible.

**Methods:**

We present a 77-year-old female patient who underwent minimally invasive surgical mitral valve replacement. After an uneventful course, the patient developed acute heart failure eleven days after surgery. Transthoracic echocardiography (TTE) revealed a new onset of TTS. The patient needed left ventricular venting and full haemodynamic flow. We successfully implanted a microaxial left ventricular assist device (Impella 5.5) using the transaxillary approach. The haemodynamic situation stabilised immediately. The patient was weaned and the Impella 5.5 was explanted after five days.

**Conclusion:**

We present the first-in-man implantation of a transaxillary Impella 5.5 in a patient with TTS. The patient benefitted from Impella 5.5 therapy with full haemodynamic support and venting of the left ventricle.

**Supplementary Information:**

The online version contains supplementary material available at 10.1186/s13019-023-02459-z.

## Backround

It can be assumed that 1–3% [1] of all patients presenting with symptoms which lead to acute coronary syndrome (ACS) have TTS. During the COVID-19 pandemic the incidence of patients presenting with ACS with subsequently confirmed TTS increased to 7.8% [2].

The incidence of cardiogenic shock (CS) in patients with TTS is estimated as ranging between 6 and 20% [3, 4]. Patients with TTS in CS have a high in-hospital mortality of 15% [5]. Both the Mayo Clinic Criteria [6] and the ESC Heart Failure Association criteria [4] are used as instruments for diagnosing TTS. They include echocardiographic evidence of hypo-, a- or dyskinesia extending beyond a single coronary vascular territory, as well as exclusion by coronary angiography of coronary atherosclerosis as a causal agent of regional wall motion abnormality. Physical or emotional triggers may additionally be present. Current evidence suggests that the pathogenesis of TTS is based on an acute surge of catecholamines through activation of the sympathetic nervous system or a result of drug therapy; it occurs in patients with an increased susceptibility of the coronary microcirculation and of cardiac myocytes to stress hormones [7–10]. The resulting downregulation of myocardial function can be understood as a protective mechanism caused by a severe reduction of perfusion.

Owing to the versatile pathogenesis and triggers of TTS, adequate and evidence-based therapy concepts are lacking; however, there is evidence that the use of catecholamines for circulatory support should be avoided, especially when patients additionally exhibit left ventricular outflow tract obstruction (LVOTO) [11–13]. As mechanical circulatory support (MCS) devices are increasingly used in patients with CS and are recommended for SCAI shock stages C, D and E [14], they are also used in patients with TTS with good results [15, 16].

The Impella 5.5 is a transvalvular, microaxial left ventricular assist device (LVAD) designed to provide circulatory support and myocardial unloading. The Impella 5.5, with its high transaortic flow capacity of 5.5 L/min, offers full flow circulatory support and, along with this, sufficient venting of the left ventricle. This generation of Impella can be implanted surgically using a transaxillary approach enabling patient mobilisation and rehabilitation.

## Case presentation

### Patient details

We present a 77-year-old female patient who was initially hospitalised due to severe primary mitral regurgitation and consequent recurrence of cardiac decompensation. The patient suffered from dyspnoea (NYHA class III). Due to calcification of the anterior and posterior mitral valve leaflet as well as an effective regurgitation orifice area (EROA) of 0.3 cm^2^ and a regurgitant volume (RV) of 55 mL as evaluated in echocardiography, an indication for minimally invasive mitral valve replacement was established.

The patient had previously suffered three non-ST-elevated myocardial infarctions (NSTEMI) in August 2019, February 2022 and October 2022, all of which were treated with drug-eluting stents (DES), two in the proximal left anterior descending artery (LAD) and one in the first diagonal branch. In addition to cardiovascular risk factors, the patient exhibited chronic heart failure with preserved ejection fraction (HFpEF). Preoperative transoesophageal echocardiography (TEE) confirmed an LVEF of 57%, LVIDd of 4.8 cm and LVIDs of 2.3 cm.

The patient underwent successful minimally invasive mitral valve replacement with a 29 mm biological prosthesis. The patient was extubated six hours after the surgery and was transferred to the normal ward on the first postoperative day (POD). The postoperative course was uneventful, a postoperative TTE confirmed a good LVEF equivalent to the preoperative level and excluded new wall motion abnormalities. On the eleventh POD, TTE showed a reduction of the LVEF to 33% and apical akinesia (Video 1). The papillary muscles of the mitral valve were visualized and found to be intact; there was also no evidence of a systolic anterior motion phenomenon of the anterior mitral valve leaflet. Emergency coronary angiography was performed which ruled out myocardial ischaemia secondary to coronary macrovascular obstruction and confirmed apical ballooning. The patient was haemodynamically impaired and decompensated, necessitating intubation and stabilisation with high-dose inotropes (vasoactive-inotropic score: 19).

The patient’s status was evaluated and the need for full circulatory support and left ventricular (LV) unloading by LV venting was confirmed. Following our evaluated standard operating procedure (SOP) for temporary MCS allocation in CS patients [17], a decision was made to implant an Impella 5.5 using a transaxillary approach within the scope of a bridge-to-recovery concept.

### Surgical procedure

**Fig. 1:**
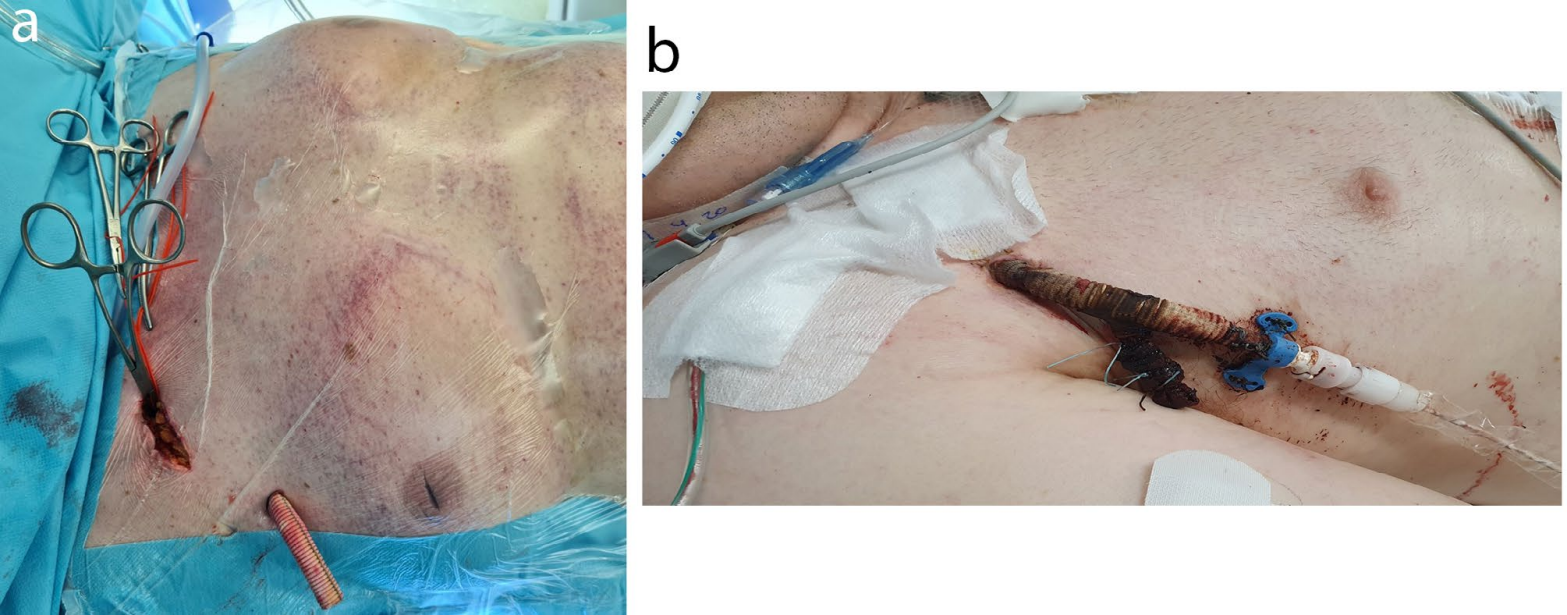
Example image of Impella 5.5 inserted through tunnelled vascular graft **(a)**. Example image of the intraoperative view of tunnelled vascular graft with clamped axillary artery **(b)**

After induction of general anaesthesia, the right axillary artery was exposed through a 6 cm incision of the skin in the infraclavicular fossa. A 10 mm HEMASHIELD vascular graft (Getinge AB, Sweden) was anastomosed to the artery, tunnelled outside the wound and diverted through the skin (Fig. 1). The Impella 5.5 device was passed through the anastomosis into the correct position in the left ventricle under fluoroscopic and echocardiographic guidance (Video 2, 3, 4).

The patient was haemodynamically stabilised and rapidly weaned off inotropic support. The patient was extubated on the first day after implantation. The duration of circulatory support on Impella 5.5 was 5 days and 18 h.

The Impella was weaned successfully. Surgical explantation of the Impella 5.5 was scheduled in the operating room on the fifth day after implantation. After removal of the Impella, the vascular graft through which the Impella was introduced into the axillary artery was shortened, ligated, and sunk under the pectoralis muscle. After three days the patient was transferred to a local hospital. Ten days after the start of MCS, TTE showed an LVEF of 55% and an LVEDD of 36 mm. Due to pre-existing coronary artery disease and to stabilize the restored pump function, heart failure therapy was established in the later course after Impella explantation. The last follow-up before discharge to outpatient follow-up took place 15 days after explantation and still showed recovery of cardiac pump function.

## Discussion

TTS is a transient wall motion abnormality. Recovery of apical dyskinesia often appears rapidly and is part of the diagnostic criteria [4].

If TTS as an acute heart failure syndrome leads to CS the patient must be bridged to cardiac recovery, which by definition is the case in all surviving TTS patients.

The challenge of managing CS in the context of TTS is that LV unloading and circulatory support with an increase in LV output should be accomplished with as few inotropic drugs as possible. In this context, MCS is an appropriate and increasingly applied treatment option that yields good results [18]. For TTS-CS patients, the Japanese InterTAK Registry describes a reduced in-hospital mortality in patients supported with MCS versus those not supported with MCS [19]. The indication for MCS in TTS-CS patient is based on the management recommendations for the SCAI Shock Stages [20]. As the complication rates under MCS increase with time on support, MCS support should be kept as short as possible. Owing to the transient character of LV dysfunction in TTS, MCS support duration in this context is relatively short [15, 18, 21]. However, there is some uncertainty as to how long MCS support should, in fact, be.

The pathophysiology of TTS and the mechanisms of different MSC devices can help in selecting the appropriate device; however, evidence for the superiority of one device over other devices is lacking. The use of intra-aortic balloon pumps (IABP) in TTS-CS is decreasing [18] due to the fact that this therapy is only able to increase the cardiac index to a limited extent and can worsen or even cause LVOTO [21]. MCS with veno-arterial extracorporeal membrane oxygenation (v-a ECMO) increases LV afterload as the circulatory support is delivered retrograde, which can cause a further increase in the already elevated LVEDP.

In general, the advantage of the microaxial LVAD is that it provides physiological cardiac support which improves coronary and end-organ perfusion while simultaneously unloading the left ventricle, thereby allowing the initiation of guideline-directed medical therapy. The positive effect of LV unloading becomes clear when comparing patients in CS treated with ECMO alone or with ECMELLA (ECMO plus Impella). LV unloading has been associated with a lower 30-day mortality [22]. MCS with ECMELLA also provides LV unloading and full circulatory support, but evidence suggests that MCS with two devices is also associated with higher complication rates [22]. Recently published case reports and series [15, 23] confirmed the pathophysiological assumed benefit of MCS support via Impella in TTS-CS and showed noteworthy survival rates and excellent myocardial recovery. These publications included cases in which Impella CP, Impella 2.5 and Impella 5.0 (only once) were used.

In our case study we were able to stabilise the patient’s hemodynamics immediately, thereby avoiding further deterioration and end-organ failure. Furthermore, haemodynamic stabilisation and weaning of inotropes prompted rhythm control and respiratory stabilisation (due to volume redistribution to the intravascular system). Our strategy of providing early MCS support with a temporary device to provide full circulatory support and immediate unloading of the impaired LV together with a short duration of MCS enabled a favourable outcome in our patient. It should be noted, however, that MCS therapy is an invasive procedure that can also lead to various complications in the long and short term [24].

### Electronic supplementary material

Below is the link to the electronic supplementary material.

Supplementary Material 1

Supplementary Material 2

Supplementary Material 3

Supplementary Material 4

Supplementary Material 5

## Data Availability

All data are available in electronic medical records.

## Glossary of Abbreviations

ECLS: extracorporeal life support
LV: left ventricle / left ventricular
LVAD: left ventricular assist device
LVEF: left ventricular ejection fraction
NYHA: New York Heart Association
TTS: Takotsubo syndrome
CS: cardiogenic shock
