# Correction: Successful treatment of cardiogenic shock due to Takotsubo syndrome with implantation of a temporary microaxial left ventricular assist device in transaxillary approach

**DOI:** 10.1186/s13019-023-02475-z

**Published:** 2024-01-08

**Authors:** Johanna K. R. von Mackensen, Ahmed El Shazly, Felix Schoenrath, Joerg Kempfert, Christoph T. Starck, Evgenij V. Potapov, Stephan Jacobs, Volkmar Falk, Leonhard Wert

**Affiliations:** 1 Department of Cardiothoracic and Vascular Surgery, Deutsches Herzzentrum der Charité – Medical Heart Center of Charité and German Heart Institute Berlin, Augustenburger Platz 1, 13353 Berlin, Germany; 2 DZHK (German Center for Cardiovascular Research) partner site Berlin, Berlin, Germany; 3 Department of Cardiothoracic Surgery, Charité − Universitätsmedizin Berlin, corporate member of Freie Universität Berlin, Humboldt-Universität zu Berlin, Berlin Institute of Health, Berlin, Germany; 4 Department of Health Sciences and Technology, ETH Zürich, Zurich, Switzerland

**Correction to: BMC Surgery (2023) 18:1**

10.1186/s13019-023-02459-z

Following publication of the original article [1], the given and family names of Ahmed El Shazly were incorrectly structured. The name was displayed correctly in all versions at the time of publication.

The original article has been corrected.
