# Supplementary material for: Successful treatment of cardiogenic shock due to Takotsubo syndrome with implantation of a temporary microaxial left ventricular assist device in transaxillary approach
Source: J Cardiothorac Surg. 2023 Nov 27;18:343. doi: 10.1186/s13019-023-02459-z (PMC10683305; doi:10.1186/s13019-023-02459-z)
Supplement: Supplementary file 5 — Supplementary Material 5 [file 13019_2023_2459_MOESM5_ESM.pdf]

Successful treatment of cardiogenic shock due to Takotsubo syndrome with implantation of a temporary microaxial left ventricular assist device in transaxillary approach

Key question

How to treat Takotsubo syndrome with cardiogenic shock in a postoperativ Setting?

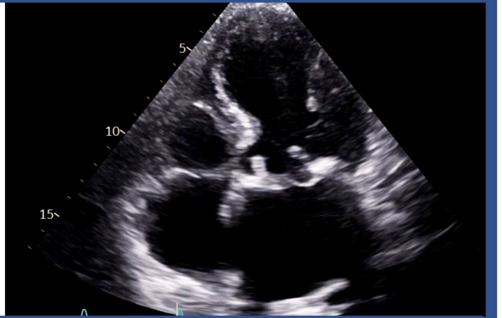

Key findings

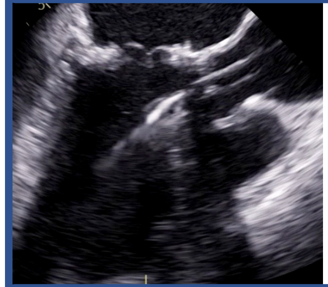

Total recovery from Cardiogenic shock due to Takotsubo Syndrome, can be achieved with Impella 5.5 as a temporary microaxial left ventricular assist device in transaxillary approach

Take-home massage

Early MCS support with the Impella 5.5 as a temporary device to provide full circulatory support and immediate unloading of the impaired LV together with a short duration of MCS can be a tailored treatment option for cardiogenic shock in Takotsubo patients

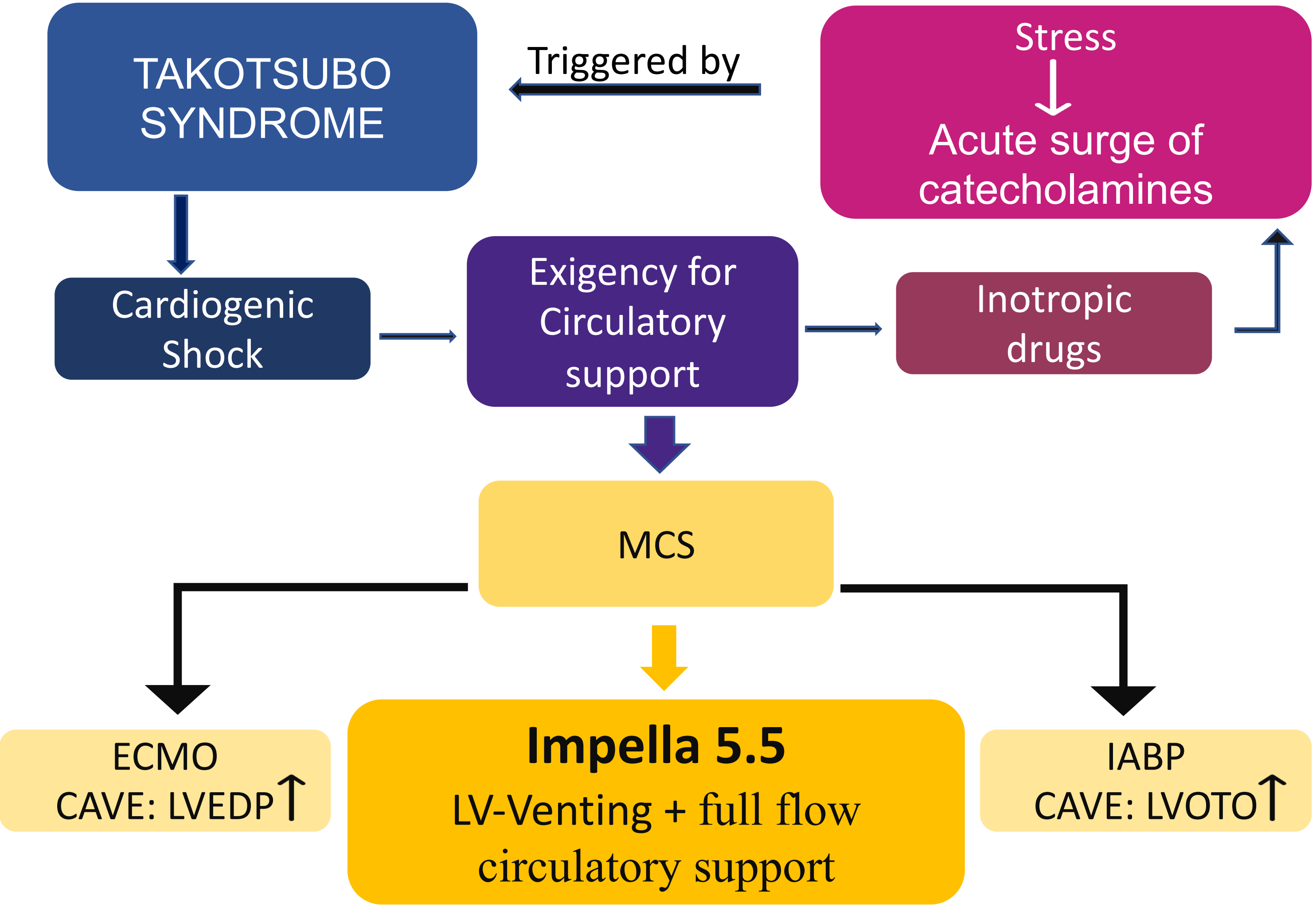

MCS= Mechanical circulatory support, LV= Left Ventricle, ECMO= Extracorporeal membrane oxygenation LVEDP= Left Ventricle end diastolic pressure, IABP= Intra-aortic balloon pump, LVOTO= left ventricular outflow tract obstruction
